# Chronic High-Level Parasitemia in HIV–Infected Individuals With or Without Visceral Leishmaniasis in an Endemic Area in Northwest Ethiopia: Potential Superspreaders?

**DOI:** 10.1093/cid/ciae002

**Published:** 2024-01-09

**Authors:** Johan van Griensven, Saskia van Henten, Aderajew Kibret, Mekibib Kassa, Hailemariam Beyene, Saïd Abdellati, Annelies de Hondt, Wim Adriaensen, Florian Vogt, Myrthe Pareyn, Koert Ritmeijer, Ermias Diro

**Affiliations:** Department of Clinical Sciences, Institute of Tropical Medicine, Antwerp, Belgium; Department of Clinical Sciences, Institute of Tropical Medicine, Antwerp, Belgium; Public Health Department, Médecins sans Frontières, Amsterdam, The Netherlands; Leishmaniasis Research and Treatment Center, University of Gondar, Gondar, Ethiopia; Public Health Department, Médecins sans Frontières, Amsterdam, The Netherlands; Department of Clinical Sciences, Institute of Tropical Medicine, Antwerp, Belgium; Department of Clinical Sciences, Institute of Tropical Medicine, Antwerp, Belgium; Department of Clinical Sciences, Institute of Tropical Medicine, Antwerp, Belgium; Department of Clinical Sciences, Institute of Tropical Medicine, Antwerp, Belgium; The Kirby Institute, University of New South Wales, Sydney, Australia; National Centre for Epidemiology and Population Health, Australian National University, Canberra, Australia; Department of Clinical Sciences, Institute of Tropical Medicine, Antwerp, Belgium; Public Health Department, Médecins sans Frontières, Amsterdam, The Netherlands; Department of Internal Medicine, University of Gondar, Gondar, Ethiopia; Department of Internal Medicine, University of Washington, Seattle, USA

**Keywords:** visceral leishmaniasis, Africa, PCR, transmission, superspreader

## Abstract

**Background:**

People with human immunodeficiency virus (PWH) with recurrent visceral leishmaniasis (VL) could potentially drive *Leishmania* transmission in areas with anthroponotic transmission such as East Africa, but studies are lacking. *Leishmania* parasitemia has been used as proxy for infectiousness.

**Methods:**

This study is nested within the Predicting Visceral Leishmaniasis in HIV-InfectedPatients (PreLeisH) prospective cohort study, following 490 PWH free of VL at enrollment for up to 24–37 months in northwest Ethiopia. Blood *Leishmania* polymerase chain reaction (PCR) was done systematically. This case series reports on 10 PWH with chronic VL (≥3 VL episodes during follow-up) for up to 37 months, and 3 individuals with asymptomatic *Leishmania* infection for up to 24 months.

**Results:**

All 10 chronic VL cases were male, on antiretroviral treatment, with 0–11 relapses before enrollment. Median baseline CD4 count was 82 cells/µL. They displayed 3–6 VL treatment episodes over a period up to 37 months. *Leishmania* blood PCR levels were strongly positive for almost the entire follow-up (median cycle threshold value, 26 [interquartile range, 23–30]), including during periods between VL treatment. Additionally, we describe 3 PWH with asymptomatic *Leishmania* infection and without VL history, with equally strong *Leishmania* parasitemia over a period of up to 24 months without developing VL. All were on antiretroviral treatment at enrollment, with baseline CD4 counts ranging from 78 to 350 cells/µL.

**Conclusions:**

These are the first data on chronic parasitemia in PWH from *Leishmania donovani*–endemic areas. PWH with asymptomatic and symptomatic *Leishmania* infection could potentially be highly infectious and constitute *Leishmania* superspreaders. Xenodiagnosis studies are required to confirm infectiousness.

## Teaser

People with HIV in northwest Ethiopia with asymptomatic and symptomatic *Leishmania* infection displayed chronic high-level parasitemia for up to 37 months. These individuals could be highly infectious and constitute *Leishmania* superspreaders. Xenodiagnosis studies are required to confirm infectiousness.

Visceral leishmaniasis (VL) is a neglected tropical disease transmitted by the bites of sandflies. In East Africa and the Indian subcontinent, VL is caused by parasites belonging to the *Leishmania donovani* species, with anthroponotic transmission [1, 2]. In the Mediterranean region and Latin America, VL is caused by *Leishmania infantum* and transmission is predominantly zoonotic. Manifestations of VL include persistent fever, splenomegaly, and pancytopenia. Without treatment, VL is usually fatal [1]. According to the World Health Organization (WHO), around 30 000 cases are estimated to occur annually at the global level [3]. East Africa currently has the highest VL burden globally.

The ambition of VL elimination in East Africa has recently been stated by WHO [4]. To inform an elimination strategy, a good understanding of which individuals are most likely to transmit the disease is essential. For most infectious diseases, infectiousness is largely driven by small subgroups, sometimes referred to as superspreaders [5, 6]. People with human immunodeficiency virus (PWH) have been put forward as potentially important sources of infection [7, 8].

Even while on antiretroviral treatment (ART), PWH generally show a decreased parasitological response to VL treatment, with a subgroup of patients manifesting with repeated VL relapses [9, 10]. Thus, there are concerns that such patients could remain infectious for many years. Whether PWH can be infectious without displaying clinical VL manifestations (ie, individuals with asymptomatic *Leishmania* infection [ALI]) is not well characterized for *L. donovani*–endemic areas. However, this could have huge implications, as such individuals would not seek VL care and could constitute hidden reservoirs of VL transmission.

In France, a chronic form of VL was described in 2010 in a case series of 10 PWH with multiple VL relapses followed up for up to 13 years [11]. Polymerase chain reaction (PCR) and blood culture results indicated continuous parasite circulation despite treatment and secondary prophylaxis, both during asymptomatic periods and the recurrent symptomatic VL episodes. This condition was labeled as active chronic VL—chronic due to the multiple VL episodes over many years, and active due to the continuous blood parasite circulation. In France, VL is caused by *L. infantum*; human immunodeficiency virus (HIV) coinfection rates are low; and, like in all *L. infantum*–endemic areas, humans are generally only considered to play a minor role in transmission. In contrast, East Africa is marked by high rates of HIV coinfection, with around 20% of people with VL coinfected with HIV in some parts of Ethiopia [9], and transmission is anthroponotic. Whether chronic parasitemia or active chronic VL also occurs in *L. donovani*–endemic areas, where it could have far-reaching implications, is not known.

In the Predicting Visceral Leishmaniasis in HIV-Infected Patients (PreLeisH) study, 490 PWH were followed up for up to 2 years in a VL-endemic area in northwest Ethiopia, with the aim to identify early predictors of VL [12]. This study also provided a unique opportunity to monitor parasite circulation in PWH over time. In this case series, we report on 10 PWH with chronic VL, defined as those developing at least 3 VL episodes during follow-up. They represent 29% of all those developing VL in the PreLeisH study. Additionally, we report on 3 HIV-coinfected individuals with ALI. Both chronic VL and ALI cases displayed high levels of parasites in their peripheral blood for up to 37 months. These are the first data ever reported on chronic parasitemia in PWH from *L. donovani*–endemic areas, providing important information for VL elimination efforts.

## METHODS

### Study Design, Set-up, and Population

This is a case series nested within the PreLeisH prospective cohort study [12], conducted between October 2017 and May 2021 in Abdurafi health center in the Amhara region, northwest Ethiopia. In the PreLeisH study, 490 PWH free of VL at the time of enrollment were followed up for up to 24 months. This included individuals with or without previous VL episodes. Individuals developing VL during the study period (incident VL cases) were followed up until the end of the study.

In this case series, we included all participants from the PreLeisH study who developed at least 3 VL episodes (defined as chronic VL) during follow-up in the study, to identify individuals most likely to be potentially infectious for a prolonged period. The 9 cases with 2 VL episodes during the study were hence not included in this case series. In addition, we included all 3 participants with ALI who displayed high parasitemia throughout the follow-up period, despite not having any VL episode before or during the study.

### Study Procedures

A study visit was planned each time the study participants presented for their routine HIV consultation (usually every 3–6 months) and in between these scheduled visits if patients presented with symptoms compatible with VL. In addition to a full clinical evaluation, blood and urine samples were collected. For the first VL episode, *Leishmania* tests were also collected weekly until the end of treatment. After each VL episode, follow-up visits were scheduled at 6 and 12 months.

Study participants were evaluated for VL at each study visit and, in case of clinical suspicion (fever >2 weeks with weight loss and/or splenomegaly), referred for routine diagnostic workup. VL diagnosis was based on microscopic examination of spleen or bone marrow aspirates [13], and grading of the parasite load was done as reported before [14]. Treatment of VL relied on liposomal amphotericin B (AmBisome) infusions and oral miltefosine combination therapy for 28 days, or longer if indicated [15]. A VL treatment episode was defined as any follow-up visit at which VL treatment was started based on the routine VL diagnostic workup. Study-specific *Leishmania* tests such as *Leishmania* PCR, urine antigen, and serology results besides the rK39 rapid diagnostic test (RDT) were not available to the physician at the time of the clinical visit. At the end of treatment, a parasitological test of cure was done by microscopy on tissue aspirates.

### Laboratory Assays and Quality Control

*Leishmania* tests were done at each study visit. This entailed *Leishmania* serological tests including the direct agglutination test (DAT; Institute of Tropical Medicine, Antwerp, Belgium), rK39 RDT (IT LEISH, Bio-Rad, Hercules, California), and rK39 enzyme-linked immunosorbent assay (ELISA) (Serion *Leishmania* IgG ELISA, Serion Diagnostics, Würzburg, Germany). Tests were executed as reported before [16]. The rK39 RDT was reported as positive or negative. For the rK39 ELISA test, results were interpreted as follows: values <10 U/mL were negative, 10–15 U/mL were borderline positive, and ≥15 U/mL were positive. A DAT titer ≥1/1600 was used to define positive tests, in line with previous studies [16].

In addition, 2 parasitological tests were done: real-time PCR targeting kinetoplast DNA (kDNA) on whole blood and a *Leishmania* urine antigen test (KAtex, Kalon Biological Ltd, Guilford, United Kingdom). The KAtex test is a semi-quantitative urine assay, with 3 levels of agglutination: 1+: weakly positive; 2+: moderately positive; 3+: strongly positive. Detection of *Leishmania* kDNA by quantitative PCR (qPCR) was done as described before [17, 18]. DNA was isolated from 300 μL whole blood using the Maxwell 16 LEV Blood DNA purification extraction kit (Promega, Leiden, The Netherlands) with the automated Maxwell 16 Instrument (AS1000, Promega). The reaction was run on a Rotor-Gene Q instrument (Qiagen, Venlo, The Netherlands). Results were expressed in cycle threshold (Ct) values. CD4 counts were done using the BD FACSCount (Becton Dickinson), typically every 6 months. The full blood count, CD4 count, and the rK39 RDT were performed locally at Abdurafi health center. Samples for DAT, rK39 ELISA, KAtex, and PCR were transported in cold chain boxes to the Leishmaniasis Training and Research Center in Gondar, Ethiopia for storage and further analysis in batch. In line with previous publications [16, 18, 19], ALI was defined as at least 1 of the *Leishmania* tests being positive in an individual without symptoms/signs of VL such as persisting fever with weight loss and splenomegaly.

### Data Collection and Quality Control

The PreLeisH study was conducted in a clinical research network that has been conducting VL clinical research in Ethiopia for over a decade, with support from the Clinical Trials Unit and the Clinical Reference Laboratory of the Institute of Tropical Medicine, Antwerp, Belgium. Study-related information was collected using a clinical and a laboratory paper case report form and subsequently transcribed into electronic case report forms, using the Macro data capture system. Data quality was monitored throughout the study, including on-site monitoring visits by the Clinical Trials Unit.

### Statistical Analysis

Only descriptive analysis was done. Continuous variables were summarized using median and interquartile range (IQR). Categorical variables were summarized using frequencies and proportions. Statistical analysis was done using Stata version 15 software (StataCorp, College Station, Texas).

### Ethical Considerations

The study was approved by the institutional/ethics review board of the Institute of Tropical Medicine, the University of Antwerp, the University of Gondar, the Amhara Regional Health Bureau, Médecins sans Frontières, and the Ethiopian research ethics review committee.

## RESULTS

### Characteristics of Study Participants

Among the 490 PWH followed up in the PreLeisH study, 34 developed VL during the study. Of these, 19 (56%) had multiple VL episodes during follow-up and 10 (29%) had 3 or more VL episodes over a period of up to 37 months. These 10 cases were included in this article and are referred to as chronic VL cases.

All participants were male, with a median age of 30 years (IQR, 28–37 years). Three were residing in the (VL-endemic) study area only temporary, 3 were stable residents having lived in the area since birth, and 4 were stable residents but born outside of the study area. One participant was a farmer, and all others were daily laborers (Table 1). ART was started at a median of 3.3 years (IQR, 1.1–6.4 years) before enrollment. The median baseline CD4 count was 82 cells/µL (IQR, 67–192 cells/µL) and <200 cells/µL for 8 participants. All but 1 had a history of VL, with a median of 2.5 (IQR, 1–8) prior episodes.

**Table 1.** Sociodemographics and History of Visceral Leishmaniasis and Human Immunodeficiency Virus (HIV) of People With HIV Included in the Case Series, Northwest Ethiopia, 2017–2021

Case	Age, y	Sex	Residence in Study Area (VL-Endemic)	Occupation	Years of HIV	Years of ART	No. of Previous VL Episodes	No. of VL Episodes in the Study	CD4 Count, Cells/µL
Chronic VL/HIV cases									
1	20	Male	Permanent, nonnative	Daily laborer	5.4	5.1	6	6	90
2	28	Male	Temporary	Daily laborer	3.6	3.6	8	5	32
3	30	Male	Permanent, nonnative	Daily laborer	6.5	6.4	3	4	50
4	31	Male	Permanent, nonnative	Daily laborer	1.3	1.1	0	4	68
5	42	Male	Temporary	Daily laborer	3.9	2.8	9	5	302
6	31	Male	Permanent, native	Daily laborer	3.1	3.1	1	4	67
7	45	Male	Permanent, native	Daily laborer	13.0	12.4	11	3	192
8	28	Male	Permanent, native	Daily laborer	1	1	2	4	458
9	37	Male	Temporary	Farmer	0.1	0.1	1	3	79
10	25	Male	Permanent, nonnative	Daily laborer	7.0	7.0	1	3	85
PWH with chronic ALI (PCR and KAtex positive)									
1	27	Male	Permanent, native	Daily laborer	0.6	0.6	0	0	350
2	37	Male	Permanent, nonnative	Daily laborer	0.2	0.2	0	0	78
3	60	Male	Permanently, native	Farmer	1.4	0.1	0	0	135

Abbreviations: ALI, asymptomatic *Leishmania* infection; ART, antiretroviral treatment; KAtex, *Leishmania* urine antigen test; PCR, polymerase chain reaction; PWH, people with human immunodeficiency virus; VL, visceral leishmaniasis.

For the 10 chronic VL cases, follow-up ranged between 12 and 37 months (median, 28 [IQR, 19–32 months]). Over this time, they had a median of 4 (IQR, 3–4; range, 3–6) VL treatment episodes, summing to a total number of experienced VL treatments (combined before and during the study) of 4 to 14 (median, 6.5 [IQR, 4–12]). For instance, case 5 had 9 episodes prior to enrollment and experienced the 10th–14th VL treatment episode during the study, whereas case 9 had 11 previous episodes and had the 12th–14th VL treatment during follow-up.

Additionally, we included 3 PWH with persistently positive results on *Leishmania* blood PCR and urine antigen test, but without a VL history and without developing disease (VL) during the 9–24 months of follow-up. They were all male, with the age ranging between 27 and 60 years. All 3 were stable residents, including 2 born in the study area. ART had been started within a year prior to enrollment, with baseline CD4 counts ranging from 78 to 350 cells/µL.

### *Leishmania* Markers Over Time

An overview of the pattern of *Leishmania* markers over time is given in Table 2 for the 5 most illustrative cases (having visits during as well as in between VL treatment episodes) and in Supplementary Table 1 for the other 5. Overall, *Leishmania* PCR blood levels were strongly positive for most of the follow-up time (Table 2), with a median Ct value of 26.0 (IQR, 23.1–30.5). This included the period before VL onset and during and between the repeated VL treatment episodes. While all displayed an increase in the Ct levels (corresponding with lower parasite levels) of the *Leishmania* PCR by the end of treatment of the first VL episode, only 4 had an undetectable *Leishmania* PCR at the end of treatment. Similarly, the urine antigen tests were strongly positive on all but a few time points, with no to minimal decreases during treatment. In general, CD4 counts were low throughout follow-up. Three participants died (cases 1, 5, and 7). For several participants (eg, case 2, 3, and 5), aiming for parasitological cure was abandoned at some point in time. Case 2 (month 18 visit), case 3 (month 28 visit), and case 5 (month M31 visit) were discharged after clinical improvement despite failing to achieve a negative test of cure; for case 2, treatment was not initiated at the subsequent visit with VL suspicion (month 27) despite a 1+ positive diagnostic tissue aspirate, as parasitological cure was not achieved at the previous visit, the general condition was fair, and starting VL treatment was not deemed to have clear clinical benefit.

**Table 2.** Overview of *Leishmania* Markers in 5 People With Human Immunodeficiency Virus With Chronic Visceral Leishmaniasis, Northwest Ethiopia, 2017–2021

Marker	Study Time Point										
**Case 1^a^**	**M0**	**M3**	**M5/Tx**	**EOT**	**M9**	**M11**	**M14**	**M17**	**M22**	**M28**	**M35**
rK39 RDT	+	+	**+**	**+**	+	+	**+**	**+**	**+**	**+**	**+**
rK39 ELISA	+	+	**+**	**+**	+	+	**+**	**+**	**+**	**+**	**+**
DAT	+	+	**+**	**+**	+	+	**+**	**+**	**+**	**+**	**+**
KAtex	3	2	**3**	**3**	0	2	**3**	**3**	**…**	**2**	**3**
PCR Ct	40.9	36.4	**27.3**	**–**	36.9	32.9	**25.6**	**27.9**	**…**	**27.5**	**25.6**
BMA	…	…	**…**	**…**	…	…	**…**	**…**	**1**	**…**	**…**
SA	0	…	**4**	**0**	0	0	**5**	**4**	**…**	**6**	**6**
CD4	90	…	**50**	**…**	63	…	**99**	**…**	**…**	**98**	**…**
**Case 2^a^**	**M0**	**M3**	**M4/Tx**	**EOT**	**M7**	**M9**	**M12**	**M18**	**M27**	**M32**	…
rK39 RDT	–	–	**–**	**–**	**–**	–	–	**–**	–	**…**	…
rK39 ELISA	–	–	**–**	**–**	**–**	–	–	**–**	–	**…**	…
DAT	+	+	**+**	**+**	**+**	+	+	**+**	+	**…**	…
KAtex	3	3	**3**	**2**	**3**	3	3	**3**	2	**…**	…
PCR Ct	37.5	31.6	**27.2**	**–**	**23.4**	31.2	24.7	**26.9**	21.3	**…**	…
BMA	0	…	**3**	**0**	**1**	…	0	**…**	1	**…**	…
SA	…	…	**…**	**…**	**…**	…	…	**6**	…	**6**	…
CD4	32	…	**19**	**…**	**12**	25	…	**7**	87	**…**	…
**Case 3**	**M0**	**M3**	**M6/Tx**	**EOT**	**M10**	**M15**	**M18**	**M21**	**M28**	…	…
rK39 RDT	+	+	**+**	**+**	+	**+**	+	**+**	**+**	…	…
rK39 ELISA	+	+	**+**	**+**	+	**+**	+	**+**	**+**	…	…
DAT	+	+	**+**	**+**	+	**+**	+	**+**	**+**	…	…
KAtex	0	2	**3**	**3**	3	**3**	3	**3**	**3**	…	…
PCR Ct	22.5	29	**19.8**	**–**	23.4	**21.8**	21.6	**22**	**19.8**	…	…
BMA	…	…	**5**	**1**	…	**4**	…	**4**	**5**	…	…
SA	…	…	**…**	**…**	…	**…**	…	**…**	**…**	…	…
CD4	50	…	**59**	**…**	102	**48**	55	**45**	**…**	…	…
**Case 4**	**M0**	**M3**	**M9/Tx**	**EOT**	**M13**	**M18**	**M19**	**M25**	**M28**	**M29**	…
rK39 RDT	–	–	**+**	**+**	–	+	**+**	**+**	+	**–**	…
rK39 ELISA	–	–	**+**	**+**	–	+	**+**	**–**	–	**–**	…
DAT	–	–	**–**	**–**	–	–	**?**	**+**	+	**+**	…
KAtex	0	0	**3**	**1**	0	3	**3**	**3**	2	**2**	…
PCR Ct	–	–	**25**	**–**	41	24	**23**	**24**	29	**24**	…
BMA	…	…	**…**	**0**	…	…	**4**	**2**	0	**1**	…
SA	…	…	**5**	**…**	…	…	**…**	**…**	…	**…**	…
CD4	68	367	**…**	**…**	116	10	**25**	**…**	…	**7**	…
**Case 5^a^**	**M0**	**M2**	**M3/Tx**	**EOT**	**M8**	**M14**	**M15**	**M31**	**M37**	…	…
rK39 RDT	+	+	**+**	**+**	**+**	+	**+**	**+**	**…**	…	…
rK39 ELISA	+	+	**+**	**+**	**+**	+	**+**	**+**	**…**	…	…
DAT	+	+	**+**	**+**	**+**	+	**+**	**+**	**…**	…	…
KAtex	0	1	**3**	**3**	**3**	3	**2**	**3**	**…**	…	…
PCR Ct	29	26.4	**25.6**	**28.3**	**0**	27	**25.6**	**22.7**	**…**	…	…
BMA	…	…	**…**	**0**	**…**	…	**5**	**5**	**…**	…	…
SA	0	…	**6**	**…**	**6**	…	**…**	**…**	**4**	…	…
CD4	302	…	**95**	**…**	**146**	167	**…**	**…**	**…**	…	…

Bolded values indicate time points with visceral leishmaniasis treatment; non-bold values indicate time points without VL treatment. Study-related *Leishmania* tests such as rK39 ELISA, DAT, *Leishmania* PCR, and urine antigen were not available to the physician at the time of the clinical visit. CD4 values are cells/μL; values for BMA & SA are parasite grade (0 if negative; 1+ to 6+); KAtex results are reported as 0 (negative), 1+, 2+ or 3+.

Abbreviations: –, negative; +, positive; BMA, bone marrow aspirate; Ct, cycle threshold value; DAT, direct agglutination test; ELISA, enzyme-linked immunosorbent assay; EOT, end of treatment; KAtex, *Leishmania* urine antigen test; M, month; PCR, polymerase chain reaction; RDT, rapid diagnostic test; SA, spleen aspirate; Tx, start of visceral leishmaniasis treatment; CD4 values are cells/µL.

^a^Individuals enrolled in the study after a negative test of cure.

Table 3 displays the *Leishmania* markers of the 3 cases with persistent ALI. While they had no VL history and VL was never diagnosed during follow-up, *Leishmania* PCR on blood and urine antigen tests were consistently and generally strongly positive during follow-up, over a period of 9–24 months. The median PCR Ct value was 24.7 (IQR, 23.0–29.5). ALI case 1 underwent bone marrow aspiration at month 24, with negative results. CD4 counts were consistently <200 cells/µL for 2 cases but not for ALI case 3. While platelets remained normal throughout follow-up, the levels of hemoglobin and white blood cells tended to be decreased, but only slightly and remained fairly stable throughout.

**Table 3.** Overview of Leishmania Markers in 3 People With Human Immunodeficiency Virus With Chronic Asymptomatic *Leishmania* Infection but Persistently Positive *Leishmania* Markers in Blood Polymerase Chain Reaction and Urine Antigen, Northwest Ethiopia, 2017–2021

Marker	Study Time Point						
**ALI case 1**	**Month 0**	**Month 3**	**Month 12**	**Month 15**	**Month 18**	**Month 21**	**Month 24**
rK39 RDT	+	+	+	…	+	…	+
rK39 ELISA	449	186	46	…	31	…	136
DAT	1/1600	1/3200	1/25 600	…	1/204 800	…	1/204 800
PCR Ct	38.6	39.5	32.3	-	29.5	…	24.9
KAtex	0	2	2	0	3	1	3
BMA	…	…	…	…	…	…	0
CD4	135	…	67	…	48	…	36
Hb	12.7	13	10.3	10.2	9.9	…	11.2
WBC	3.97	4.04	2.08	3.13	3	…	3.4
Platelets	273	285	220	274	271	…	263
**ALI case 2**	**Month 0**	**Month 3**	**Month 6**	**Month 9**	**Month 12**	…	…
rK39 RDT	+	+	+	+	+	…	…
rK39 ELISA	>800	>800	>800	>800	>800	…	…
DAT	1/204 800	1/204 800	1/204 800	1/204 800	1/204 800	…	…
PCR Ct	23.4	26.4	24.4	22.8	23	…	…
KAtex	2	2	3	3	3	…	…
CD4	78	…	144	…	82	…	…
Hb	10.7	11.3	11.3	12	9.8	…	…
WBC	3.01	3.35	6.16	4.45	3.1	…	…
Platelets	226	251	249	228	212	…	…
**ALI case 3**	**Month 0**	**Month 3**	**Month 6**	**Month 9**	…	…	…
rK39 RDT	–	–	–	–	…	…	…
rK39 ELISA	–	–	–	–	…	…	…
DAT	1/204 800	1/25 600	1/51 200	1/204 800	…	…	…
PCR Ct	25.9	24.0	22.0	21.3	…	…	…
KAtex	2	2	3	0	…	…	…
CD4	350	…	399	…	…	…	…
Hb	11	10.2	11	8.2	…	…	…
WBC	2.13	2.5	4.98	2.8	…	…	…
Platelets	173	187	208	161	…	…	…

Study-related *Leishmania* tests such as rK39 ELISA, DAT, *Leishmania* PCR, and urine antigen were not available to the physician at the time of the clinical visit. CD4 values are cells/µL; values for BMA & SA are parasite grade (0 if negative, 1+ to 6+); KAtex results are reported as 0 (negative), 1+, 2+ or 3+; platelet values represent platelet count/µL.

Abbreviations: –, negative; +, positive; ALI, asymptomatic *Leishmania* infection; BMA, bone marrow aspirate; Ct, cycle threshold value; DAT, direct agglutination test, expressed in titer (positive if titer ≥1/1600); ELISA, enzyme-linked immunosorbent assay, expressed in units/mL (positive if >15 U/mL); Hb, hemoglobin (gram/deciliter); KAtex, *Leishmania* urine antigen test; PCR, polymerase chain reaction; RDT, rapid diagnostic test; WBC, white blood cell count (cells/µL).

## DISCUSSION

To our knowledge, this is the first description of PWH from an *L. donovani*–endemic area with chronic high-level parasitemia. Markers were positive during but also between the repeated VL treatment episodes. In addition, we describe 3 PWH without VL before or during the study who displayed high levels of *Leishmania* parasitemia and antigenuria for 9–24 months, without developing VL. To the best of our knowledge, these are the first 3 such cases from both *L. infantum–* and *L. donovani*–endemic areas.

While the chronic VL cases described fit the entity of active chronic VL described in France [11], we did not perform culture and thus could not demonstrate that viable parasites could be recovered from the blood. Nevertheless, the persistent and high direct parasite markers in blood and urine indicate active replication since *Leishmania* DNA detected by PCR in the blood is degraded rapidly [20].

Xenodiagnosis studies performed in *L. infantum*–endemic areas in VL cases with HIV or other immunosuppressive conditions found they were more infectious to sandflies than immunocompetent individuals and also demonstrated that PWH with ALI were capable of transmitting the parasite [21, 22]. Xenodiagnosis studies in PWH in *L. donovani–*endemic areas—where transmission is anthroponotic—remain to be conducted, including PWH with symptomatic and asymptomatic *Leishmania* infection.

A characteristic feature of superspreaders is that, even when relatively rare, they contribute to a relatively large part of transmission [5, 6]. For example, a small number of highly infectious dogs have been shown to harbor close to 90% of *Leishmania* parasites [23]. In Ethiopia, a modeling study suggested that 3.2% of humans could contribute to 53%–79% of the infected sandfly population [24]. In northwest Ethiopia, the HIV prevalence in VL cases remains at around 20% [9, 25]. Among the 34 incident VL cases in the PreLeisH study, 10 (29%) developed at least 3 VL episodes over the 12–37 months of follow-up. Chronic VL cases could potentially be infectious for very prolonged periods, and given the repeated exposure to VL treatment, potentially also of drug-resistant parasites. These individuals are also highly mobile. This case series only included chronic VL cases (≥3 VL episodes in the study). However, several of the 9 patients—not included in this study—with 2 VL episodes during follow-up also had persistent parasitemia, so this condition could be more common.

PWH with ALI and chronically high parasitemia were rather rare. Only 3 were identified with consistent parasitemia among the 412 individuals in the PreLeisH study without VL treatment episodes and with at least 3 PCR measurements done during follow-up (ie, 0.7% of study participants). Even while few, they could constitute hidden reservoirs of VL transmission for a long time, as such individuals would not seek VL care. Further studies on the role of superspreaders are required to inform the East African VL elimination initiative [4].

While qPCR levels in peripheral blood generally correlated with infectiousness in studies from *L. infantum*–endemic areas [21, 22], recent studies have suggested that the skin is a much underappreciated site of active parasite replication [5, 23, 26]. In (symptomatic) dogs, skin qPCR parasite loads were higher, even at levels similar to the spleen [23, 27], and correlated well with infectivity during xenodiagnosis [23, 27]. To assess this in humans, it could be useful to conduct studies using skin microbiopsy devices [28], which mimic the sandfly bite, in parallel with qPCR on peripheral blood and ideally xenodiagnosis. Reliable tools for infectivity would also be needed to assess whether reducing parasite load with treatment/prophylaxis has an impact on the transmission potential. While qPCR testing is currently not available at most health facilities in East African countries, highly accurate and simple loop-mediated isothermal amplification assay has been proposed as a molecular point-of-care test adapted to resource-constrained settings [29].

Strengths of our study include that it was conducted within a well-established clinical research network, with due attention to high-standard laboratory procedures and quality control, and oversight by a Clinical Trials Unit. There are a number of important limitations to acknowledge. As mentioned, xenodiagnosis studies would have strengthened the public health relevance of our findings. More frequent visits between treatment episodes would have allowed more refined analysis of the evolution of *Leishmania* parasitemia. For the 3 individuals with ALI but high parasitemia, it remains unsure whether they developed VL after the study.

## CONCLUSIONS

We describe 10 cases of active chronic VL in PWH, displaying multiple VL episodes over a 37-month follow-up period. Both during and in between VL treatment, *Leishmania* PCR and urine antigen tests were consistently and generally strongly positive, indicating prolonged opportunities for transmission. We also report 3 cases without VL history but with persistently strongly positive *Leishmania* markers, which could act as hidden sources of infection. Both groups could act as superspreaders and constitute a major challenge for VL elimination in the area. If the infectiousness of these individuals is confirmed in future xenodiagnosis studies, simple biomarkers of infectiousness should be developed to identify the most infectious individuals, complemented by studies on interventions to reduce transmission from these individuals.

## Supplementary Data

Supplementary materials are available at *Clinical Infectious Diseases* online. Consisting of data provided by the authors to benefit the reader, the posted materials are not copyedited and are the sole responsibility of the authors, so questions or comments should be addressed to the corresponding author.

## Supplementary Material

ciae002_Supplementary_Data

## Notes

***Acknowledgments.*** The authors thank all of the study participants and the staff involved in the study at the Abdurafi health center and the Gondar Leishmaniasis Research and Treatment Center.

***Financial support.*** The PreLeisH study was funded by the Institute of Tropical Medicine (Antwerp, Belgium) , grant no. 757013.
