# Supplementary material for: Chronic High-Level Parasitemia in HIV–Infected Individuals With or Without Visceral Leishmaniasis in an Endemic Area in Northwest Ethiopia: Potential Superspreaders?
Source: Clin Infect Dis. 2024 Jan 9;79(1):240–6. doi: 10.1093/cid/ciae002 (PMC11259210; doi:10.1093/cid/ciae002)
Supplement: ciae002_Supplementary_Data [file ciae002_supplementary_data.docx]

**Table S1. Overview of Leishmania markers in five HIV infected individuals with chronic visceral leishmaniasis, North-Ethiopia 2017-2021**

| **CASE 6** |  |  |  |  |  |  |
| --- | --- | --- | --- | --- | --- | --- |
|  | **M0** | **M1/Tx** | **EOT** | **M9** | **M13** | **M20** |
| **rK39 RDT** | + | + | + | + | + | + |
| **rK39 ELISA** | + | + | + | + | + | + |
| **DAT** | + | + | + | + | + | + |
| **KATEX** | 2 | 3 | 3 | 3 | 3 | 3 |
| **PCR Ct** | 24,4 | 25,6 | 37,3 | 22,9 | 23,1 | 25,6 |
| **SA** |  | 6 | 0 | 6 | 6 | 6 |
| **CD4** | 67 |  |  | 63 | 145 | 50 |
|  |  |  |  |  |  |  |
| **CASE 7** |  |  |  |  |  |  |
|  | **M0** | **M4/Tx** | **EOT** | **M8** | **M12** |  |
| **rK39 RDT** | + | + | + |  |  |  |
| **rK39 ELISA** | + | + | - |  |  |  |
| **DAT** | + | + | + |  |  |  |
| **KATEX** | 3 | 3 | 3 | 3 | 3 |  |
| **PCR Ct** | 30,5 | 27,9 | 40,9 |  | 25,2 |  |
| **BMA** |  |  | 0 |  |  |  |
| **SA** |  | 4 |  | 6 | 6 |  |
| **CD4** | 192 | 104 |  |  | 56 |  |
|  |  |  |  |  |  |  |
| **CASE 8 ^a^** |  |  |  |  |  |  |
|  | **M0** | **M6/Tx** | **EOT** | **M18** | **M24** | **M30** |
| **rK39 RDT** | + | + | + | + | + |  |
| **rK39 ELISA** | + | + | + | + | + |  |
| **DAT** | + | + | + | + | + |  |
| **KATEX** | 3 | 3 | 3 | 3 | 3 |  |
| **PCR Ct** | 39,4 | 27,5 | 42,3 | 23,3 | 21,9 |  |
| **BMA** | 0 |  |  |  |  |  |
| **SA** |  | 6 | 0 | 6 | 6 | 6 |
| **CD4** | 458 | 154 |  | 201 |  |  |
|  |  |  |  |  |  |  |
| **CASE 9** |  |  |  |  |  |  |
|  | **M0** | **M10/Tx** | **EOT** | **M19** | **M23** |  |
| **rK39 RDT** | + | + | + | + |  |  |
| **rK39 ELISA** | + | + | + | + |  |  |
| **DAT** | + | + | + | + |  |  |
| **KATEX** | 0 | 3 | 3 | 3 |  |  |
| **PCR Ct** | 41,5 | 20,3 | 35,2 | 22,8 |  |  |
| **BMA** |  | 3 |  | 2 |  |  |
| **SA** |  |  | 0 |  | 6 |  |
| **CD4** | 79 |  |  | 55 |  |  |
|  |  |  |  |  |  |  |
| **CASE 10 ^a^** |  |  |  |  |  |  |
|  | **M0** | **M3/Tx** | **EOT** | **M8** | **M14** |  |
| **rK39 RDT** | + | + |  | + |  |  |
| **rK39 ELISA** | + | + |  | + |  |  |
| **DAT** | - | + |  | + |  |  |
| **KATEX** | 2 | 3 | 3 | 3 |  |  |
| **PCR Ct** | 29,7 | 21,6 | 30,5 | 19.4 |  |  |
| **BMA** | 0 | 4 | 0 |  |  |  |
| **SA** |  |  |  | 5 | 6 |  |
| **CD4** | 85 |  |  | 49 |  |  |

**^a^** individuals enrolled in the study after a negative test of cure (TOC)

**Grey** areas indicate time points with VL treatment; **White** areas indicate time points without VL treatment. Study related *Leishmania* tests such as rK39 ELISA, DAT, *Leishmania* PCR and urine antigen were not available to the physician at the time of the clinical visit.

RDT: rapid diagnostic test; ELISA: enzyme-linked immunosorbent assay; DAT: direct agglutination test; PCR: polymerase chain reaction; SA: spleen aspirate; BMA: bone marrow aspirate; Tx: start of VL treatment; EOT: end of treatment; Ct: PCR cycle threshold value; VL: visceral leishmaniasis.
